# Modulating Drug Release from Gastric-Floating Microcapsules through Spray-Coating Layers

**DOI:** 10.1371/journal.pone.0114284

**Published:** 2014-12-03

**Authors:** Wei Li Lee, Jun Wei Melvin Tan, Chaoyang Nicholas Tan, Say Chye Joachim Loo

**Affiliations:** School of Materials Science and Engineering, Nanyang Technological University, 50 Nanyang Avenue, 639798 Singapore, Singapore; University of South Florida, United States of America

## Abstract

Floating dosage forms with prolonged gastric residence time have garnered much interest in the field of oral delivery. However, studies had shown that slow and incomplete release of hydrophobic drugs during gastric residence period would reduce drug absorption and cause drug wastage. Herein, a spray-coated floating microcapsule system was developed to encapsulate fenofibrate and piroxicam, as model hydrophobic drugs, into the coating layers with the aim of enhancing and tuning drug release rates. Incorporating fenofibrate into rubbery poly(caprolactone) (PCL) coating layer resulted in a complete and sustained release for up to 8 h, with outermost non-drug-holding PCL coating layer serving as a rate-controlling membrane. To realize a multidrug-loaded system, both hydrophilic metformin HCl and hydrophobic fenofibrate were simultaneously incorporated into these spray-coated microcapsules, with metformin HCl and fenofibrate localized within the hollow cavity of the capsule and coating layer, respectively. Both drugs were observed to be completely released from these coated microcapsules in a sustained manner. Through specific tailoring of coating polymers and their configurations, piroxicam loaded in both the outer polyethylene glycol and inner PCL coating layers was released in a double-profile manner (i.e. an immediate burst release as the loading dose, followed by a sustained release as the maintenance dose). The fabricated microcapsules exhibited excellent buoyancy in simulated gastric fluid, and provided controlled and sustained release, thus revealing its potential as a rate-controlled oral drug delivery system.

## Introduction

Even with the recent advancements in drug delivery, oral administration still remains the most preferred route due to its ease of administration, avoidance of pain and versatility in drug formulation [1]. However, conventional oral dosage forms (e.g. tablets, capsules) do have limitations. These include poor control over release rates [2] and a lack of complete absorption of drugs due to variable and short gastrointestinal (GI) transit time [3]. As such, in order to maintain drug plasma level over prolonged durations, frequent dosing is required. High pill burden leads to poor compliance to medication, and consequentially poor treatment outcomes, side effects, and medication errors – all potentially life threatening scenarios.

Floating dosage forms with prolonged gastric residence time (GRT) have therefore been developed to increase bioavailability, and reduce dosing frequency by providing controlled and sustained release capabilities [3], [4]. Unlike a single-unit floating system (e.g. hydrodynamically balanced system (HBS) tablet), which is unreliable in prolonging the GRT owing to its ‘all-or-nothing’ emptying process, multiple-unit floating dosage forms (e.g. hollow microcapsules) avoid the vagaries of gastric emptying and reduce the risk of local irritation [5]. While the drug release from HBS is generally governed by the dissipation of hydrated boundary gel layers [6], microcapsules offer greater versatility in controlling the release of drugs with different water solubilities through the manipulation of capsule parameters, such as shell thickness, polymer types and specific localization of drugs. In contrast to the conventional gas-generating floating devices [5], hollow microcapsules allow for instant buoyancy upon contact with gastric fluid, without any lag time in buoyancy. Floating microcapsules can be fabricated through an emulsion solvent removal technique [7], in which high compression forces and elevated temperatures are not required, unlike other common methods of producing floating tablets [8].

While conventional oral drug delivery devices generally delivers a single drug only, a drug carrier that encapsulates multiple drugs alleviates the need for numerous oral tablets (pill burden), especially for diseases that require treatment with multiple therapeutic agents, such as chronic cardiovascular diseases, tuberculosis, Parkinson’s disease, etc. However, developing such an oral delivery system is challenging, as drug-drug interactions should be avoided and controlling the simultaneous release of hydrophilic, hydrophobic and amphiphilic drugs from a delivery system is non-trivial. Sato et al. developed hollow microspheres (i.e. microballoons) composed of enteric acrylic polymers by the emulsion solvent diffusion method [2]. The amount of riboflavin released can be modulated by incorporating hydrophilic polymers such as hydroxypropylmethylcellulose into the shell matrix of these microballoons. However, drug and hydrophilic polymer embedment within the shell caused pore formation, which would decrease their buoyancy arising from rapid water ingress. In another example, metformin HCl, metoprolol tartrate and fenofibrate encapsulated within a hollow, buoyant microcapsule had been shown to yield undesirably slow release of hydrophobic fenofibrate [7]. Slow and incomplete release of hydrophobic drug reduces its absorption, leading to wastage, poor bioavailability and low pharmacological responses [9]. Since floating microspheres exhibit prolonged GRT of 5 h in humans [10], [11], a complete and sustained release of drugs that are mainly absorbed in the upper GI tract within this period would clearly be advantageous [12]–[14]. These factors therefore require a radical drug formulation design that prolongs GRT, while able to tune the simultaneous release of drugs with different hydrophilicities.

Coating has been widely applied on drug dosage forms, and its usage is in extending drug release [15], [16], increasing density of drug carriers for high density gastric retention systems [17], overlaying with bioadhesives [18], or providing protection to achieve site-specific delivery of oral drugs [19]. For instance, Tang et al. demonstrated that extended release of hydrophilic drugs from alginate beads was achieved by applying an acid resistant Eudragit coating through dip coating [16]. Furthermore, Krishnamachari et al. reported that pre-synthesized polyester cores that were microencapsulated in Eudragit polymer, released drugs only when the enteric coating dissolved at pH above 7 [20]. However, reports on drug-loaded layer coating onto floating microcapsules and its drug release kinetic are scant. In this paper, a surface-coated microcapsule system was presented to incorporate hydrophobic drug into the coating layers with the aim of controlling its release profile, and achieving practically useful release rates. Spray-coating, the coating technique used in this work, has considerable promise as a scalable deposition technique for high-throughput large-area coverage by proper choice of the atomizing pattern and computer-controlled spray coaters [21], [22]. The first objective was to develop a spray-coated, gastric-floating, controlled-releasing microcapsule system that is capable of complete, simultaneous release of two drugs with different properties, i.e. hydrophilic metformin HCl and hydrophobic fenofibrate. Design parameters for coating, such as number of layers, layer morphology and polymer types, would be explored to tune the drug release profiles. The second objective was to use this spray-coating technique to develop a double-profile drug releasing system of a single hydrophobic piroxicam; whereby a burst release as the loading dose, followed by a sustained release as the maintenance dose. A loading dose is most useful for piroxicam with long systemic half-lives [23].

## Materials and Methods

### 2.1 Materials

Poly(L-lactide) (PLLA, intrinsic viscosity (IV): 2.38, Bio Invigor), poly(caprolactone) (PCL, molecular weight (MW): 10 kDa, Aldrich), polyethylene glycol (PEG, MW: 10 kDa, Sigma-Aldrich) and poly(vinyl alcohol) (PVA, MW: 30–70 kDa, Sigma–Aldrich) were used without further purification. Drugs (i.e. metformin HCl, fenofibrate and piroxicam), Cremophor EL and n-hexane were purchased from Sigma-Aldrich. Dichloromethane (DCM) was purchased from Tedia Company Inc. Olive oil (Pietro Coricelli) was used. Phosphate-buffered saline (PBS) at pH 7.4 was from OHME, Singapore. All drugs and solvents were used as received, unless otherwise noted. The simulated gastric fluid (SGF) (pH 1) was prepared by adding 0.1 M HCl solution (Merck) and 0.02% (w/v) Tween 20 (Tokyo Kasei). The simulated intestinal fluid (SIF) contained pH 6.8 phosphate buffer and 0.02% (w/v) Tween 80 (Sigma-Aldrich).

### 2.2 Encapsulation of free drug into hollow microcapsules

For encapsulation, metformin HCl (0.1 g) was first dissolved in 1 mL of PVA aqueous solution (0.1% w/v). The drug solution was then introduced dropwise into PLLA-PCL solution (0.3 g PLLA, 0.1 g PCL dissolved in 5 ml of DCM), followed by the addition of olive oil (0.01 mL) under magnetic stirring. The resultant mixture was added to 50 mL of PVA aqueous solution (0.25% w/v) containing 1 mL of DCM and emulsified under overhead stirring (250 rpm) for 4 min at room temperature (25°C). With the formation of emulsion droplets, the emulsion was quickly transferred to rotary evaporator with the addition of PVA solution (150 mL) to solidify the microcapsules. The microcapsules produced were centrifuged, rinsed with deionized water, lyophilized and stored in a desiccator. For the fabrication of fenofibrate-loaded microcapsules, fenofibrate was either dissolved in PLLA-PCL solution (fenofibrate embedded within the capsule shell) or suspended in 1 mL of PVA aqueous solution (0.1% w/v) (fenofibrate in the hollow cavity). Non-drug-loaded hollow microcapsules were fabricated using the same method except 1 mL of PVA aqueous solution (without drug added) was introduced into PLLA-PCL solution.

### 2.3 Spray-coating on microcapsules

Pre-fabricated microcapsules (0.1 g) placed on a mesh were spray-coated using MediCoat DES 1000 Benchtop Coating System (Sono-TekCorp., Milton, NY) within a clean hood. The microcapsules were coated in a row-by-row sweeping manner. Power for the frequency generator was set at 1.4 W. Air shroud level to coat sample F1 (fenofibrate) was 4 psi, while air shroud level of samples F2 and F3 (fenofibrate) and samples P1, P2 and P3 (piroxicam) were at 2 psi. Parameters used to achieve different configurations of the coated microcapsules are listed in **Table 1**. The coated microcapsules were dried in a vacuum oven at 40°C for 1 day.

**Table 1:** Parameters used to achieve different configurations of the coated microcapsules.

	Coating Layer 1		Coating Layer 2		Coating Layer 3	
Sample	Solution	Flow Rate(mL/min)	Solution	Flow Rate(mL/min)	Solution	Flow Rate(mL/min)
F1	PCL (0.1 g)	1	-	-	-	-
	Feno (0.014 g)					
	DCM (1 mL)					
F2	DCM (0.5 mL)	0.5	PCL (0.05 g)	0.2	-	-
			Feno (0.014 g)			
			DCM (0.5 mL)			
F3	DCM (0.5 mL)	0.5	PCL (0.05 g)	0.2	PCL (0.1 g)	0.2
			Feno (0.014 g)		DCM (1 mL)	
			DCM (0.5 mL)			
			PEG (0.1 g)			
P1	DCM (0.5 mL)	0.5	Pirox (0.014 g)	0.2	-	-
			DCM (1 mL)			
			PCL (0.2 g)			
P2	DCM (0.5 mL)	0.5	Pirox (0.082 g)	0.2	-	-
			DCM (2 mL)			
			PCL (0.2 g)		PEG (0.1 g)	
P3	DCM (0.5 mL)	0.5	Pirox (0.082 g)	0.2	Pirox (0.021 g)	0.2
			DCM (2 mL)		DCM (1 mL)	

### 2.4 Morphological analysis

The surface and internal morphologies of the microcapsules were viewed under scanning electron microscopy (SEM, JEOL JSM-6360A) at 5 kV. Prior to analysis, samples were first mounted onto a metal stub and cross-sectioned approximately at the centre line using a razor blade. Samples were then coated with gold using a sputter coater (SPI-Module). Measurement of capsule size (in diameter) was performed on the SEM images using the ImageJ software.

### 2.5 Determination of actual drug loading

For the determination of actual loading of water-soluble metformin HCl, 10 mg of microcapsules (n = 3) were first dissolved in 1 mL of DCM. Extraction of the hydrophilic drug was then achieved with the use of 10 mL PBS. The concentration of metformin HCl in aqueous solution was analyzed using UV-Vis spectrophotometer (Shimadzu UV-250), at the wavelength of 233 nm. To determine loading of hydrophobic fenofibrate and piroxicam, microcapsules were first dissolved in 1 mL of DCM. Subsequently, n-hexane (5 mL) was added to precipitate polymers and other drugs (if any). The mixture was centrifuged and the supernatant was dried. SGF (10 mL) containing 2% w/v Cremophor EL was then added to dissolve the solid fenofibrate or piroxicam for UV-Vis analysis at 292 nm and 335 nm, respectively.

### 2.6 In vitro buoyancy test

The buoyancy of the microcapsules was tested through a visual observation method [11], [16], [18]. For each microcapsule group, 50 individual microcapsules, in triplicate, were placed into 20 mL SGF filled in the vials (20 mL volume capped bottle). The bottles were incubated in a water bath at 37°C under magnetic stirring at 250 rpm for 24 h [16]. At each pre-determined time, the number of floating microcapsules was counted visually. The percentage of floating microcapsules (an indication of buoyancy) was calculated according to the ratio of the number of floating microcapsules to the total number of microcapsules.

### 2.7 Drug release study

Metformin HCl *in vitro* release study was carried out in SGF and SIF. For poorly water-soluble fenofibrate and piroxicam, the release test was conducted in the medium with the addition of 2% w/v Cremophor EL to maintain a sink condition [24]. Microcapsules (20 mg) were placed, in triplicate, in vials containing 20 mL dissolution medium and were maintained at 37°C with a magnetic rotation speed of 250 rpm. At prescribed time points, medium (1 mL) from each vial was removed and replaced with fresh medium. The drug content was analyzed using UV-Vis spectrophotometer (λ_metformin HCl_ = 233 nm, λ_fenofibrate_ = 292 nm, λ_piroxicam in SGF_ = 335 nm, λ_piroxicam in SIF_ = 361 nm).

### 2.8 Statistical analysis

Data from different sets of samples were compared by unpaired Student's t-test. Differences were considered statistically significant when P≤0.05.

## Results and Discussion

### 3.1 Formation of spray-coated, hollow microcapsules

Gastric-floating hollow microcapsules were prepared using water/oil/water (W_1_/O/W_2_) emulsion solvent evaporation method. In order to achieve a hollow structure, solvent extraction rate has to be manipulated. The nascent emulsion droplets of capsules are formed under overhead stirring. Subsequently, during the hardening process, the solvent removal rate can be accelerated through the use of rotary evaporator under reduced pressure. As a result, an increased removal rate of solvent (i.e. DCM) from the emulsion droplets to the external aqueous phase reduces the time allowed for any coalescence of inner water droplets (W_1_) with the external water phase (W_2_). This would give rise to a large hollow cavity within the microcapsule.

**Figure 1:**
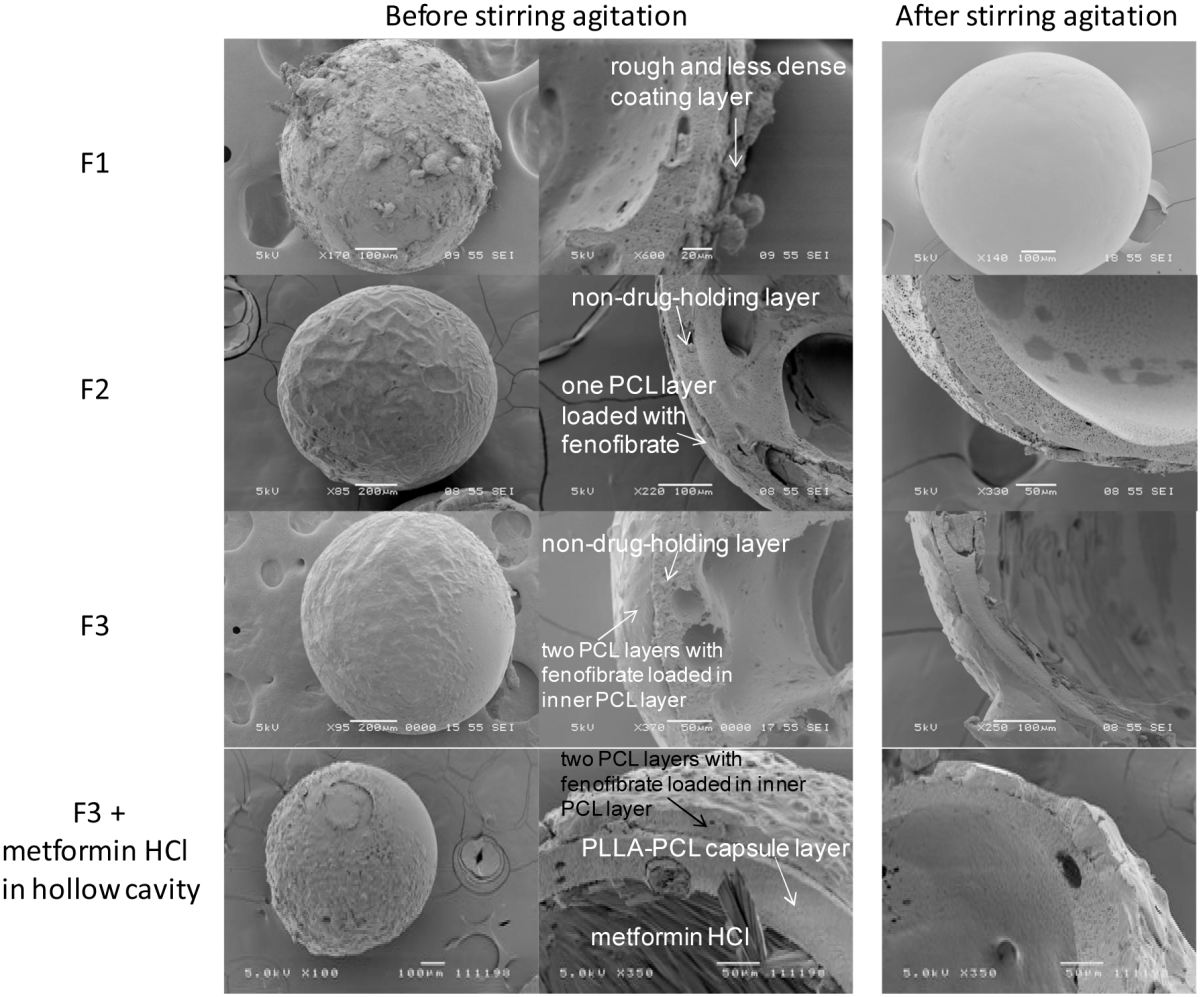
External and internal morphologies of the coated microcapsules with fenofibrate loaded in PCL coating layer before and after stirring agitation in SGF for 24 h. Row 1: F1; Row 2: F2; Row 3: F3; Row 4: F3 coating configuration together with metformin HCl encapsulated within the hollow cavity. Since F3 exhibited two PCL coating layers, the coating layer in F3 was observed to be thicker than F2, which only exhibited one coating layer.

Spray-coating was employed to coat the drug-loaded polymer layer onto the surface of the pre-fabricated microcapsule, using MediCoat DES 1000 Benchtop Coating System. Different coating polymers and configurations were achieved under the conditions listed in **Table 1**. Coating of well-defined drug-containing layers on the microcapsule and maintaining its structural integrity during agitation in SGF (similar to gastric peristaltic activity) are certainly not trivial. Microcapsules (F1) coated using the solution of fenofibrate, PCL and DCM exhibited a rough and less dense coating layer around the capsules (**Figure 1**, row1), unlike the non-coated microcapsules that were observed to be smooth (**Figure S1**). However, with agitation in SGF, this coating layer on microcapsule F1 fragmented and peeled off (**Figure 1**).

To overcome this peeling-off effect and achieve a denser layer, DCM (0.5 mL) was first sprayed onto the surface of the microcapsules before spray-coating with polymer and drug. DCM would partially dissolve the surface, creating an adhesive interface for the subsequent polymer-drug coating. At the same time, the air shroud pressure for spray-coating was lowered from 4 psi to 2 psi. The function of the air shroud (nitrogen gas) is to produce a uniformly distributed flow of air around the nozzle’s atomizing surface. When the atomized spray drops is continuously being sprayed onto the microcapsules, the increase in air shroud induces higher downward pressure that may blow off the droplets already deposited on the capsules and cause the displacement of the semi-solid coating layer, leading to a less dense layer. Therefore, the air shroud pressure should be optimized to create a dense layer. In addition, the flow rate of the polymer solution was reduced, i.e. from 1.0 mL/min to 0.2 mL/min. Since the capsules were coated in a row-by-row sweeping manner, the reduction in flow rate would allow a polymer to solidify layer-by-layer during multiple rounds of spray-coating. Morphological study (**Figure 1**, row 2) shows that by altering these parameters, the fenofibrate-loaded PCL coating layer for sample F2 was relatively dense and remained intact after 24 h of agitation. This manipulated coating method was similarly employed to coat multiple layers (F3) on microcapsules. Multiple coating layers of higher thickness were observed to exhibit dense morphology and to remain intact after SGF agitation (**Figure 1**, row 3).

### 3.2 Layer-coated microcapsules that sustain release of both hydrophilic and hydrophobic drugs

**Figure 2:**
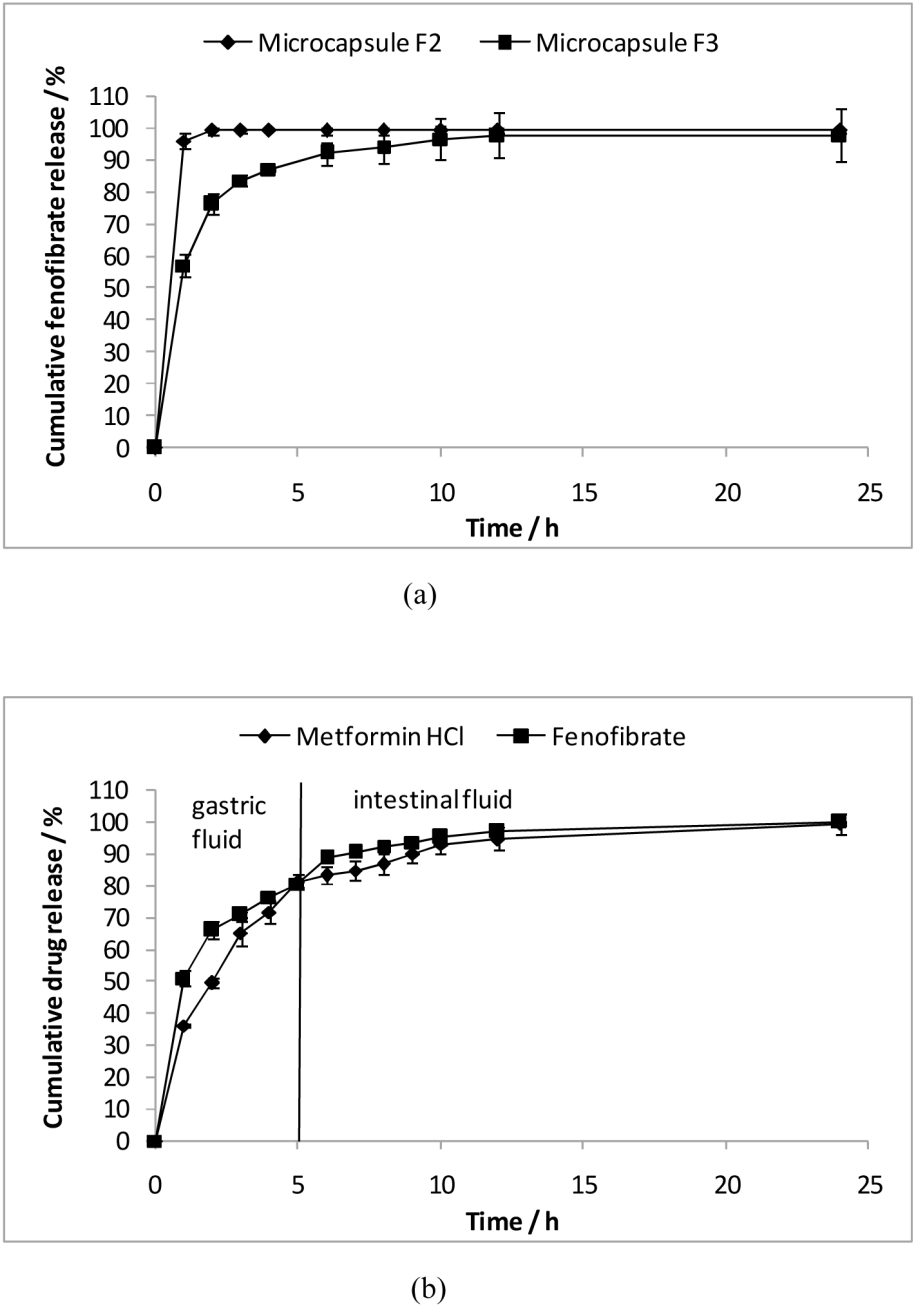
Drug release profiles of spray-coated gastric-floating microcapsules. (a) Release profiles of fenofibrate from microcapsules F2 and F3 in SGF. (b) Release profiles of metformin HCl and fenofibrate from microcapsules in SGF for 5 h followed by release into SIF at 37°C. It should be noted that the release profile of each drug from microcapsules had been derived from the microcapsules that co-encapsulated two different drugs.

Biodegradable polymers in PLLA, PCL and their blends were used to fabricate surface-coated, hollow microcapsules. These polymers were chosen because of their biocompatibility and manipulability to achieve different drug release profiles. The release of hydrophobic fenofibrate was found to be impractically slow (20–30% drug release in 24 h) when either encapsulated within the hollow cavities, or embedded within the 25 wt% PCL/75 wt% PLLA capsule shell. (**Figure S2**). Fenofibrate’s partition coefficient for [octanol]/[water] is as high as 1.5×10^5^ [25]. The dissolution process of fenofibrate occurs with great difficulty due to its neutral and hydrophobic nature. This phenomenon indicates the importance of substantial amount of medium uptake required for fenofibrate release. The PLLA-PCL shell of the microcapsule limits the rate of SGF influx and retards the release of fenofibrate. Fenofibrate, being hydrophobic, has strong affinity for larger fraction of hydrophobic PLLA with high glass transition temperature and increased crystallinity [26], which acts as an additional hindrance to its release rate. In order to accelerate the release of hydrophobic fenofibrate to practically useful rates, it was hypothesized that coating of a rubbery layer onto the microcapsule would be able to address this. A fenofibrate-loaded layer coated on blank microcapsule reduces drug diffusion distance, while the use of rubbery PCL polymer promotes drug dissolution and diffusion. In the drug release study, it was noticed that the release of fenofibrate from sample F2 (a fenofibrate-loaded PCL coating layer on blank microcapsule) was massively accelerated, exhibiting a 100% burst release within 1 h (**Figure 2a**). At drug release condition of 37°C, PCL chains (with a very low glass transition temperature of 60°C) are in a highly flexible state with increased free volume [27], which enables better dissolution of the hydrophobic drug, thus increasing release rate. An additional non-drug-holding PCL layer (sample F3, **Figure 1**- row 3) was therefore further coated onto the fenofibrate-loaded PCL layer to suppress this burst (p<0.05 up to 8 h when comparing F2 and F3). This outermost non-drug-holding layer served as an additional diffusion barrier, thus limiting initial burst, yet sustaining fenofibrate release for up to 8 h (**Figure 2a**).

**Figure 3:**
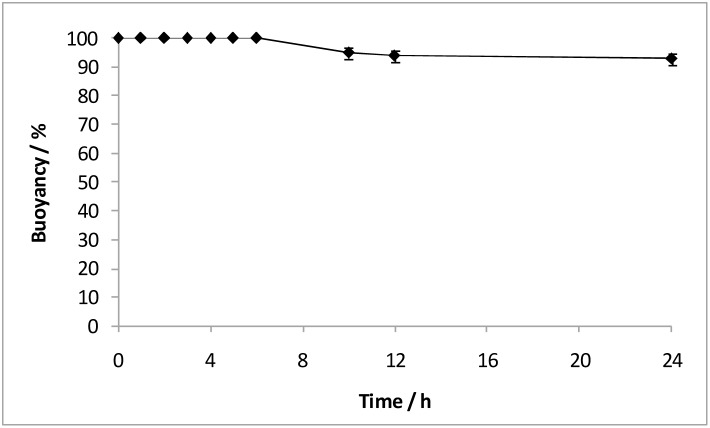
Percent buoyancy vs time profile of the coated microcapsules containing metformin HCl and fenofibrate. Metformin HCl was encapsulated within the hollow cavity of PLLA-PCL microcapsules, with fenofibrate incorporated into the PCL coating layer. The hollow cavity and non-drug-holding PLLA-PCL capsule layer loaded with olive oil gave rise to excellent buoyancy.

To realize a dual-drug-loaded floating delivery system, metformin HCl was encapsulated within the hollow cavity of PLLA-PCL microcapsules, with fenofibrate incorporated into the coating layer, based on the coating configuration of F3 (**Figure 1**, row 4**)**. The buoyancy profile of these microcapsules were excellent (**Figure 3**), capable of floating continuously over 24 h. Such floating capabilities could be attributed to the low density provided by the hollow cavity, and the hydrophobic nature of the non-drug-holding PLLA-PCL capsule layer loaded with olive oil [7]. The absence of drug localized within the PLLA-PCL capsule layer prevents the formation of pores upon drug release, which would promote water influx into the hollow cavity and decrease the buoyancy [2]. It is worth mentioning that the partial coverage of the PCL coating layers on microcapsules did not impair their buoyancy. The size of microcapsule was 691±97 µm, and cross-sectioned examination showed dense coating layer, with free metformin HCl encapsulated within the hollow cavity of the microcapsules. The actual loading of metformin HCl and fenofibrate were 5.14±0.21 wt% and 3.26±0.05 wt%, respectively. It is noted that hydrophilic metformin HCl can be easily dissolved in water, thus allowing for direct encapsulation (with high or adjustable content) into the capsules. In addition, delivery devices with prolonged GRT would increase the bioavailability of drugs, thus requiring a lower dosage [28], [29]. The release profiles of metformin HCl and fenofibrate from the coated microcapsules for 5 h in SGF (pH 1) followed by release in SIF (pH 6.8) at 37°C are shown in **Figure 2b**. Multiple drugs, each with different hydrophilicities, were observed to be released from the microcapsules in a sustained manner for around 7 h. The absence of metformin HCl within the cavities of the microcapsules after 24 h concurs with its complete release (**Figure 1**, row 4). When metformin HCl was encapsulated within the hollow cavities, the non-drug-holding PLLA-PCL (75∶25 wt%) capsule layer acted as a rate-limiting barrier that suppresses initial burst while controlling release rate of metformin HCl. Hydrophobic and semi-crystalline PLLA retards appreciable medium influx and significantly limits drug diffusion. In contrast to pure PLLA, the addition of rubbery PCL into PLLA matrix results in a less dense and more rubbery capsule layer [27], [30], which moderates the diffusion of metformin HCl. The PCL coating layers on the microcapsules, on the other hand, controlled the release of fenofibrate from the coating layer, which was similarly observed for sample F3. The specific localization of drugs in different compartments (i.e. coating layers and hollow cavity) allows two drugs with distinct water solubilities to be released in a controlled manner, thus providing synergistic and multiple strengths of a single spray-coated microcapsule. Morphological view of microcapsule (**Figure 1**, row 4) shows that the capsule and coating layers still remained unchanged and intact after 24 h, suggesting the diffusional drug release [31]. *M*_t_/*M*_∞_ = *kt* ^0.43^ is used to describe the simple diffusion-controlled release [32], where *M*_t_/*M*_∞_ is the cumulative fraction of drug release, *t* is time and *k* is the constant characteristic of diffusion. It is well-known that a power law could approximate the Fickian diffusion from the sphere geometry, with an exponent of 0.43 [33]. The plot of the metformin HCl release data in SGF for cumulative release percentage versus time^0.43^ shows a linear profile (R^2^>0.99) (**Figure S3**), suggesting the simple-diffusion controlled release of metformin HCl that had diffused into the outer layer upon water ingress [33], [34]. The release data demonstrates that certain amount of drugs would be released in the stomach depending on the gastric residence time of microcapsules (∼5 h) while the remaining drugs would be released in the proximal intestinal region [23], [29]. Since the main absorption site of metformin HCl and fenofibrate was reported to be upper GI tract [12], [14], their complete release in the upper GI tract within 5–7 h would clearly be advantageous.

### 3.3 Layer-coated microcapsules achieving continuous loading and maintenance release doses

By virtue of the versatility and robustness of the coating layers, multiple layering using different polymers can provide a means to control drug release rates. The spray-coating technique established in this paper was employed to coat piroxicam-loaded polymer layers onto non-drug-loaded hollow microcapsules (**Table 1**, P1 to P3). The role of non-drug-loaded hollow microcapsules (substrate) is to provide the buoyancy, whereas the coating layers on these microcapsules are used to deliver the drug in a controlled manner. For the delivery of piroxicam (with long elimination half-life ∼30 to 86 h), a combination of two different drug release kinetics is required, i.e. a burst release upon dosage administration for the loading dose and a sustained release for the maintenance dose. The initial loading dose provides the amount of the drug to reach therapeutic levels rapidly while a low maintenance dose keeps the amount of the drug in the body at the appropriate therapeutic level. The inclusion of both loading and maintenance doses into a single microcapsule protects drugs from premature degradation, provides a continuous controlled release profile, and aids in improving patient compliance.

**Figure 4:**
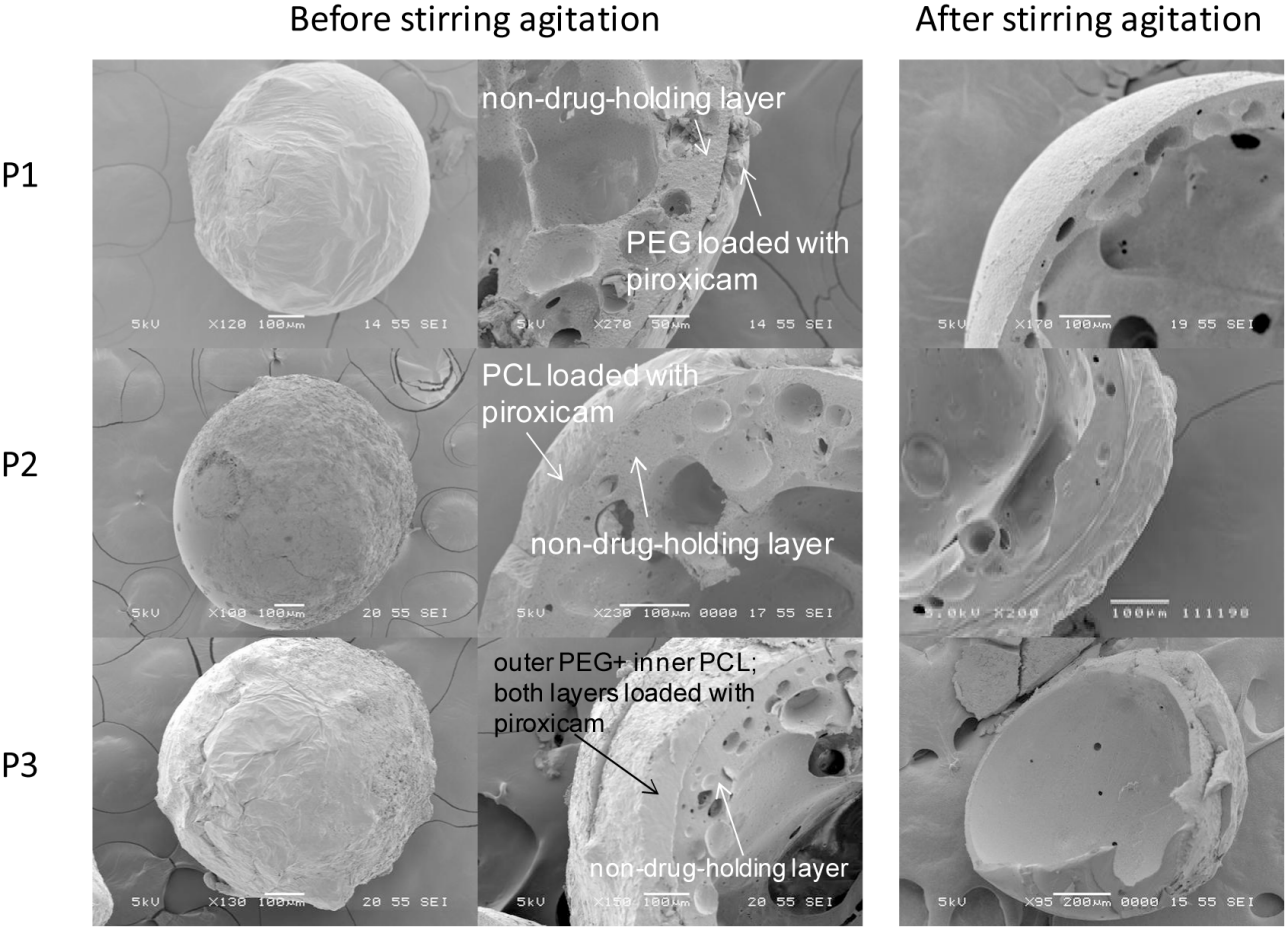
External and internal morphologies of the coated microcapsules with piroxicam loaded in coating layers before and after stirring agitation in SGF for 24 h. Row 1: P1 (PEG coating layer); Row 2: P2 (PCL coating layer); Row 3: P3 (outer PEG and inner PCL coating layers).

**Figure 5:**
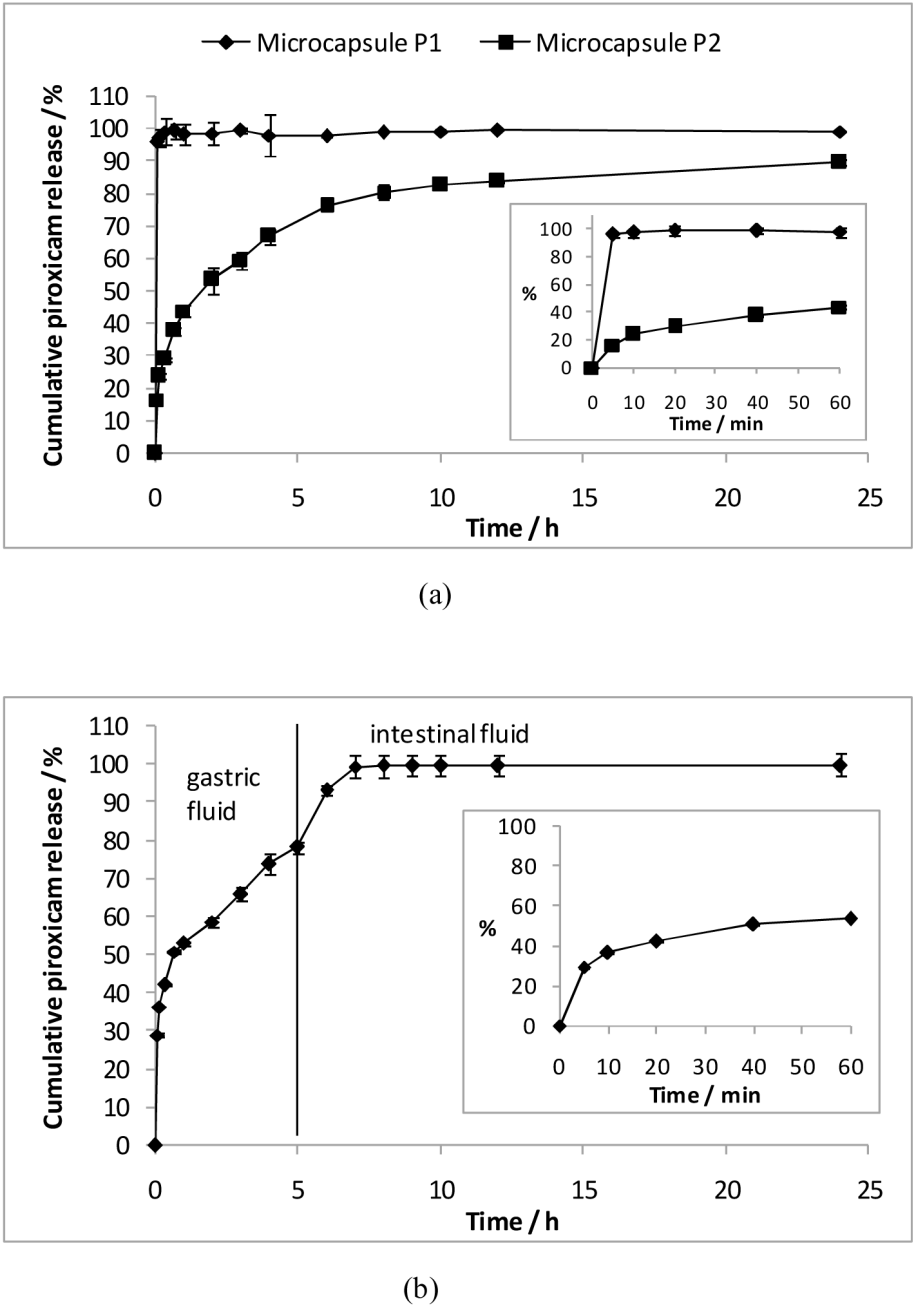
Piroxicam release profiles of spray-coated gastric-floating microcapsules. (a) Release profiles of piroxicam from microcapsules P1 and P2 in SGF. (b) Release profile of piroxicam from microcapsule P3 in SGF for 5 h followed by release into SIF at 37°C. The insets are the close-up views of the release within first 60 min in SGF.

**Figure 6:**
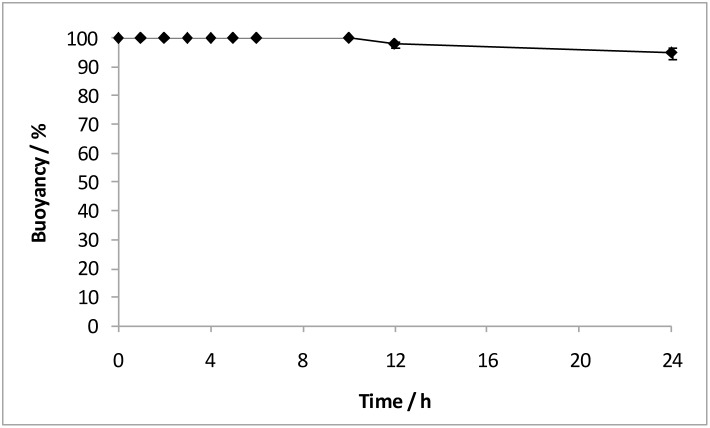
Percent buoyancy vs time profile of the coated microcapsules containing piroxicam (P3). Piroxicam was incorporated into both the outer PEG and inner PCL coating layers. The hollow cavity and non-drug-holding capsule layer loaded with olive oil gave rise to excellent buoyancy.

Two sets of coated microcapsules (P1 and P2), as shown in **Figure 4**, were first fabricated to study the individual release kinetics of hydrophobic piroxicam. The parameters for coating are listed in **Table 1**. Drug release study shows that microcapsule P1 with a piroxicam-loaded PEG coating layer exhibited a complete release within 5 min in SGF (**Figure 5a**). The rapid dissolution of hydrophilic PEG coating layer led to a huge burst release upon quick SGF influx, as evidenced by a smooth external surface and the absence of the coating layer after immersion in SGF (**Figure 4** **,** row 1). Microcapsule P2, which had a piroxicam-loaded PCL coating layer, yielded a sustained release for 10 h (p<0.01 when comparing P1 and P2) (**Figure 5a**). The PCL coating layer still remained intact after 24 h (**Figure 4** **,** row 2), suggesting the diffusional drug release. Subsequently, microcapsule P3 was fabricated by combining the inner coating layer of piroxicam-loaded PCL and the outermost coating layer of piroxicam-loaded PEG. The total piroxicam loading of microcapsule P3 was found to be 14.98±0.24 wt%. The amount of piroxicam in each coating layer was determined based on the dosages reported by Joseph et al., where loading dose and maintenance dose are ∼25% and ∼75% of total dosage, respectively [23]. Buoyancy test shows that the coated microcapsules containing piroxicam (P3) remained afloat for 24 h (**Figure 6**). The *in vitro* drug release study to mimic drug release condition along the GI tract is shown in **Figure 5b**. Approximately 30% of piroxicam was released within the first 5 min as a result of complete PEG dissolution, serving as a loading dose. It is noteworthy that the loading dose (burst release at 5 min) is adjustable by simply manipulating the amount of drug loaded in PEG layer. Subsequently, a continuous release (∼50%) in SGF for 5 h and another 2 h for complete release into SIF were observed for PCL coating layer; both serve as the maintenance dose. Piroxicam, which is used to manage osteoarthritis, may potentially induce damage to the walls of the final part of the digestive system, i.e. cecum, colon, rectum [35]. At the same time, erratic elimination of piroxicam occurs in some elderly patients, resulting in a high fluctuation in the steady state levels [36]. Therefore, the complete and sustained release (∼7 h) of piroxicam in the upper GI tract would certainly be desirable [35].

## Conclusions

A spray-coating technique was demonstrated to layer drug-loaded polymers onto floating microcapsules. The coated microcapsules exhibited excellent buoyancy in simulated gastric fluid. Controlled and sustained release of both hydrophilic metformin HCl and hydrophobic fenofibrate was achieved by embedding the hydrophobic drug within the rubbery PCL coating layer, while at the same time encapsulating hydrophilic drug within the hollow cavity. This spray-coating technique was further implemented to develop floating microcapsules that deliver two continuous dosages, i.e. loading and maintenance doses. Hydrophobic piroxicam was loaded into both the outer PEG and inner PCL coating layers. From this study, we determined that by designing and tailoring the coating configuration, hydrophobic drugs can be loaded, and their release profiles can be fine-tuned accordingly. Therefore, this coated microcapsule system may be a promising platform to deliver single or multiple drugs orally, while providing controlled and sustained release.

## Supporting Information

Figure S1 **SEM image of cross-sectional view of microcapsules before coating.** Multiple microcapsules are shown to demonstrate homogeneity of the capsule morphology.
(TIF)

Figure S2 **Non-coated 25 wt% PCL/75 wt% PLLA microcapsules containing fenofibrate.** SEM images of cross-sectional view of 25 wt% PCL/75 wt% PLLA microcapsules, whereby (a) free fenofibrate was attached onto the inner wall of the microcapsule, and (b) fenofibrate was embedded within the capsule shell. (c) Release profiles of fenofibrate from 25 wt% PCL/75 wt% PLLA microcapsules encapsulating free fenofibrate within the hollow cavities, or within the capsule shell.
(TIF)

Figure S3 **Plot of cumulative metformin HCl release (%) versus time^0.43^.** A linear profile with R^2^>0.99 is shown.
(TIF)
