# Supplementary figures and images for: Modulating Drug Release from Gastric-Floating Microcapsules through Spray-Coating Layers
Source: PLoS One. 2014 Dec 3;9(12):e114284. doi: 10.1371/journal.pone.0114284 (PMC4254999; doi:10.1371/journal.pone.0114284)

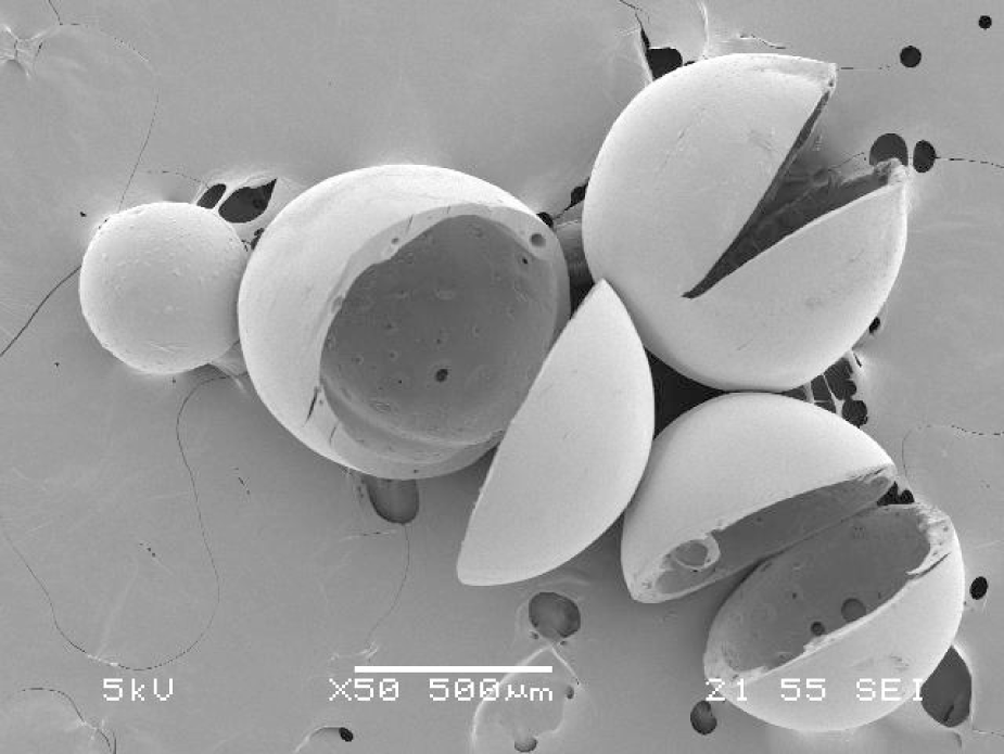

Supplement: Figure S1 — SEM image of cross-sectional view of microcapsules before coating. Multiple microcapsules are shown to demonstrate homogeneity of the capsule morphology. (TIF) [file pone.0114284.s001.tif]

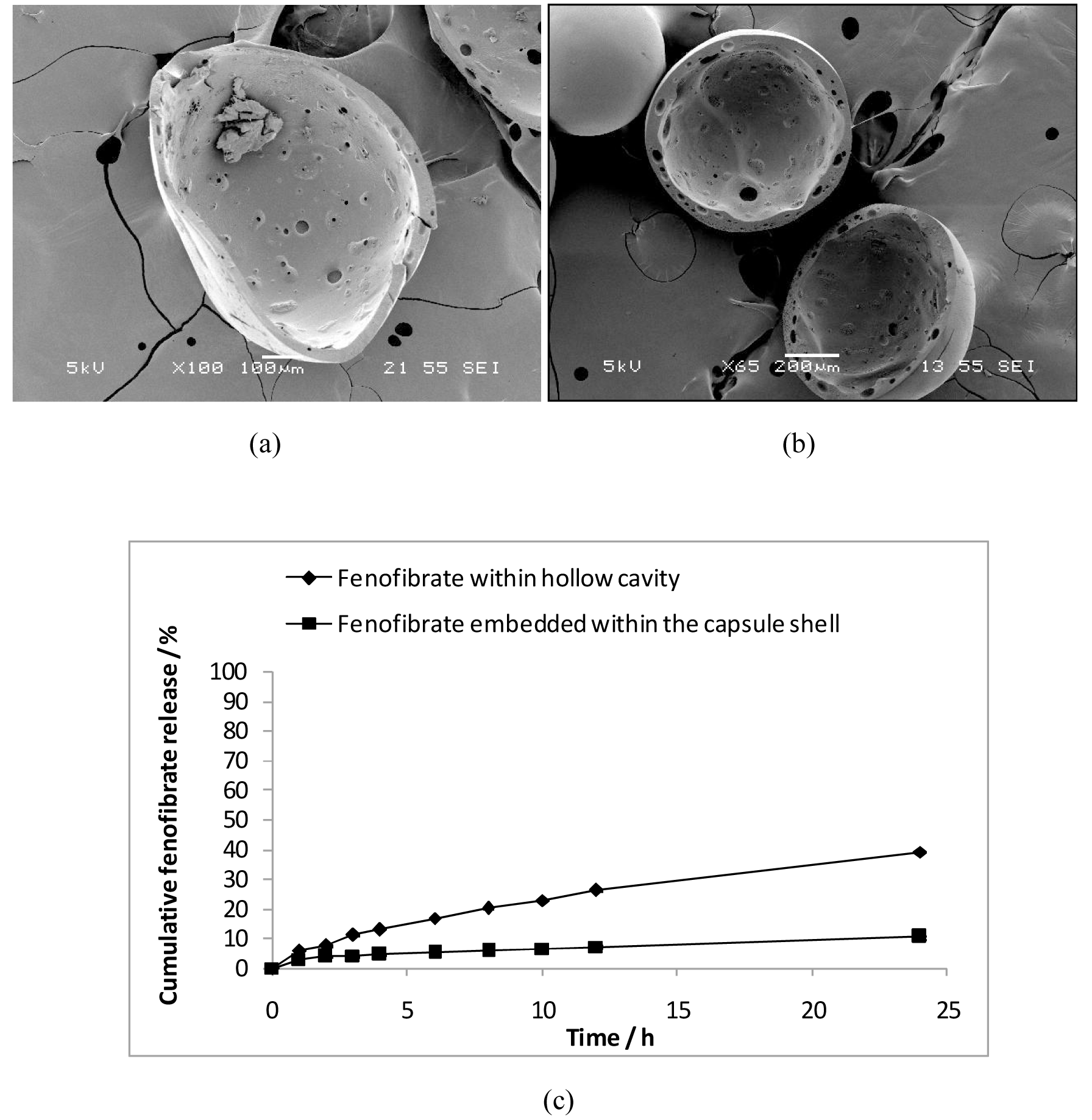

Supplement: Figure S2 — Non-coated 25 wt% PCL/75 wt% PLLA microcapsules containing fenofibrate. SEM images of cross-sectional view of 25 wt% PCL/75 wt% PLLA microcapsules, whereby (a) free fenofibrate was attached onto the inner wall of the microcapsule, and (b) fenofibrate was embedded within the capsule shell. (c) Release profiles of fenofibrate from 25 wt% PCL/75 wt% PLLA microcapsules encapsulating free fenofibrate within the hollow cavities, or within the capsule shell. (TIF) [file pone.0114284.s002.tif]

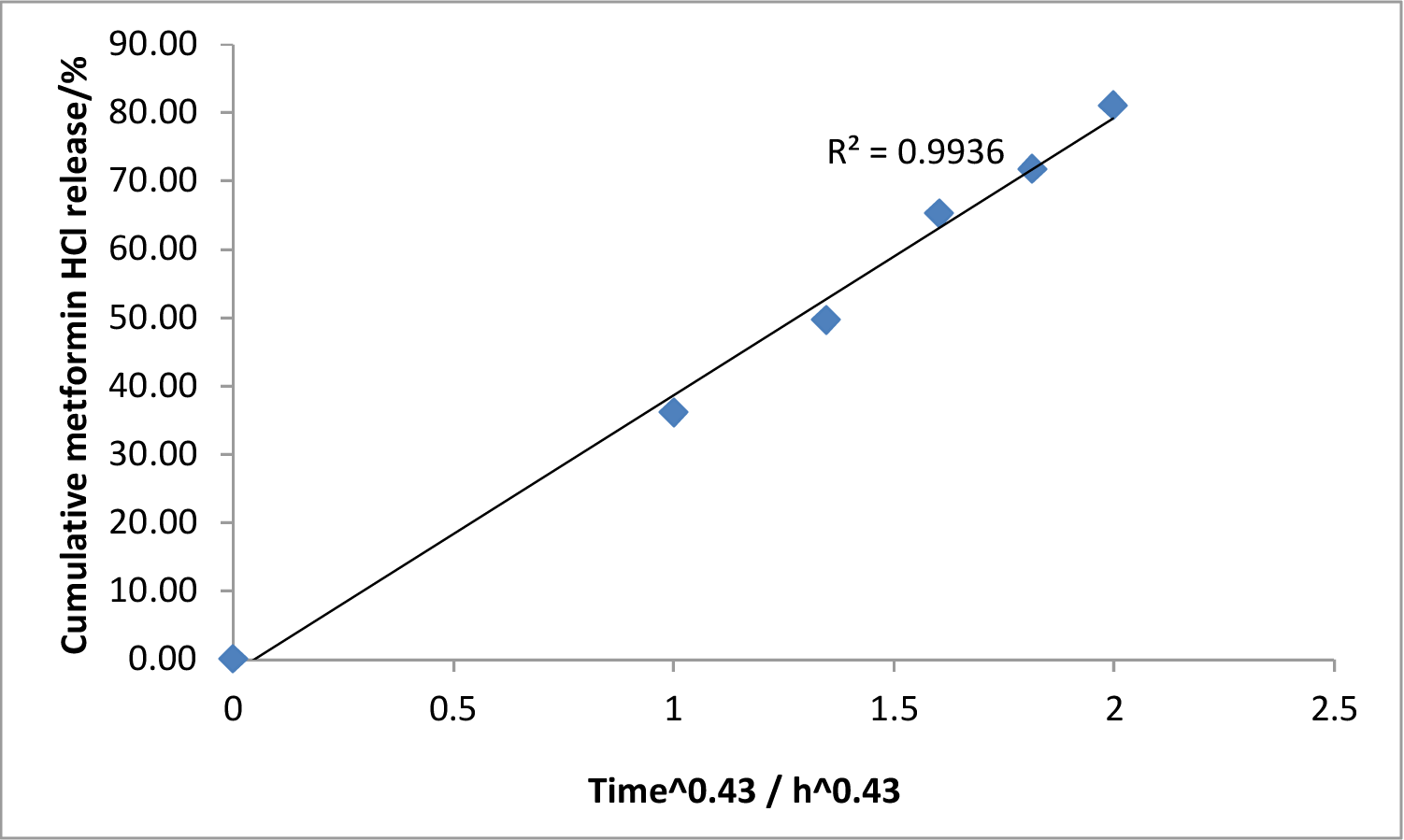

Supplement: Figure S3 — Plot of cumulative metformin HCl release (%) versus time0.43. A linear profile with R2>0.99 is shown. (TIF) [file pone.0114284.s003.tif]
